# Long Leukocyte Telomere Length at Diagnosis Is a Risk Factor for Dementia Progression in Idiopathic Parkinsonism

**DOI:** 10.1371/journal.pone.0113387

**Published:** 2014-12-12

**Authors:** Sofie Degerman, Magdalena Domellöf, Mattias Landfors, Jan Linder, Mathias Lundin, Susann Haraldsson, Eva Elgh, Göran Roos, Lars Forsgren

**Affiliations:** 1 Department of Medical Biosciences, Umeå University, Umeå, Sweden; 2 Department of Pharmacology and Clinical Neuroscience, Umeå University, Umeå, Sweden; 3 Department of Statistics, Umeå University, Umeå, Sweden; 4 Department of Community Medicine and Rehabilitation, Umeå University, Umeå, Sweden; Okayama University Graduate School of Medicine, Dentistry and Pharmaceutical Sciences, Japan

## Abstract

Telomere length (TL) is regarded as a marker of cellular aging due to the gradual shortening by each cell division, but is influenced by a number of factors including oxidative stress and inflammation. Parkinson's disease and atypical forms of parkinsonism occur mainly in the elderly, with oxidative stress and inflammation in afflicted cells. In this study the relationship between blood TL and prognosis of 168 patients with idiopathic parkinsonism (136 Parkinson's disease [PD], 17 Progressive Supranuclear Palsy [PSP], and 15 Multiple System Atrophy [MSA]) and 30 controls was investigated. TL and motor and cognitive performance were assessed at baseline (diagnosis) and repeatedly up to three to five years follow up. No difference in TL between controls and patients was shown at baseline, nor any significant difference in TL stability or attrition during follow up. Interestingly, a significant relationship between TL at diagnosis and cognitive phenotype at follow up in PD and PSP patients was found, with longer mean TL at diagnosis in patients that developed dementia within three years.

## Introduction

The telomere structures at the chromosome ends are essential for cell growth and survival by providing genomic stability. They consist of repetitive (TTAGGG) DNA sequences and associated telomere binding proteins, serving as a buffer of noncoding DNA [1]. Due to the incomplete replication of linear DNA (the “end replication problem”), telomeres shorten by each cell division and telomere length is therefore regarded as a biomarker for cellular aging. However, telomere length homeostasis is affected by numerous genetic and environmental factors, including telomerase activity, oxidative stress and chronic inflammation [2]. Parkinson's Disease (PD), Multiple System Atrophy (MSA) and Progressive Supranuclear Palsy (PSP) are age-related disorders which are idiopathic in the vast majority of cases. Clinical onset is typically in the elderly and oxidative stress due to increased production of free radicals or reduced antioxidative capacity has been implicated in disease processes [3].

Studies on telomere length in the most common neurodegenerative disorders, Alzheimer's disease (AD) and PD show conflicting results (reviewed in [4]). In PD telomere length has been investigated mainly in blood but in one study also in substantia nigra. Studies from Japan reported higher proportion of short leukocyte telomeres in males with PD compared to healthy controls [5], but higher proportion of long telomeres in females [6]. A non-significant trend for shorter telomeres in PD compared to controls was reported from Germany [7]. These studies are based on fairly small populations but four larger studies have also reported on telomere length. No significant difference was found between PD patients and controls in a Finnish study [8] – with slightly longer telomeres in the PD group. In studies from the US with blood samples collected prior to the onset of PD the quartile with the shortest telomeres was three times less likely to develop PD (96 patients and 172 age-matched controls) compared with the quartile with longest telomeres [9], and another study (408 patients and 809 matched controls; only males) also found short telomeres to be associated with reduced risk for PD [10]. Finally, a study from England reported significantly longer mean telomere lengths in leukocytes in the PD group compared to matched controls [11]. In a smaller subgroup telomere length in the substantia nigra was investigated and no difference was found between the PD and controls [11]. Thus, results on telomere length as a contributing factor for the risk to develop PD are conflicting and most studies find an association between long mean telomeres and having a PD diagnosis or developing PD, which is a counterintuitive association given the knowledge of telomeres for aging and as a target for cellular oxidative stress and inflammation [12].

To our knowledge no studies on telomere length in PD has assessed variation of leukocyte telomere lengths in blood repeatedly collected in patients over several years. Furthermore, the possible role of telomere lengths as a prognostic factor is unknown. Therefore we have investigated telomere length in a community-based study population with idiopathic parkinsonism and in matched controls followed three to five years from the time of diagnosis with the aims to investigate if telomere lengths differ between patients with PD, with atypical parkinsonism (MSA, PSP), and controls. Further we correlated telomere length with severity of motor and cognitive performance and survival in order to evaluate the prognostic relevance of telomere length in idiopathic parkinsonism.

## Materials and Methods

### Participants

The patients participated in the NYPUM-project (NY [new] Parkinsonism in UMeå), a prospective population-based study on idiopathic parkinsonism in the local catchment area of Umeå University Hospital in northern Sweden with around 142 000 inhabitants. Only patients with previously undiagnosed idiopathic parkinsonism were included in the study and the inclusion period was between January 1, 2004 to April 30, 2009. In total 185 patients with idiopathic parkinsonism were identified during the period and blood was donated from 172 of these (93%). Patients were followed with yearly reassessment of diagnosis by two movement disorder specialists, and neuropsychological testing at diagnosis and after one, three and five years. A control group of 30 persons matched to age and sex of the first 50 included patients were investigated at baseline and after three and five years. Patients have been followed up for four to nine years (except those who died prior to four years) and the diagnoses in the study refer to that of the last follow-up. The presynaptic dopamine system was examined with 123I-N-(omega)-fluoropropyl-2-beta-carbomethoxy-3-beta-(4-iodophenyl)-nortropane single-photon emission computed tomography (FP-CIT SPECT) in patients and controls at baseline. All controls and two patients had normal scans. These two patients plus two patients that could not be classified as PD, MSA or PSP were excluded from the study and the remaining 168 patients with pathological scans were included. All samples at baseline were collected prior to treatment initiation by dopaminergic drugs.

Written, informed consent was obtained from all participants. The study was approved by the Ethics Committee of the Faculty of Medicine at Umeå University.

### Clinical assessments

The diagnostic criteria applied were the UK Parkinson's Disease Society Brain Bank criteria (UKPDSBB) for PD [13], the NINDS-SPSP criteria for PSP [14], the Gilman criteria for MSA [15]. To measure the severity of parkinsonism patients were assessed with the Unified Parkinson's Disease rating Scale (UPDRS I–III) and the Hoehn and Yahr staging [16]. Cognitive performance in PD patients was classified as normal, mild cognitive impairment (MCI) according to the Movement Disorder Task Force guidelines level I [17] and dementia according published criteria [18]. The MCI classification was based on a battery of neuropsychological tests that were assessed at baseline, one, three and five years; for details of the test battery, see Domellöf et. al. [19]. In cases that did not perform the full neuropsychological battery, the MCI classification was based on result on the Mini Mental State Examination (MMSE) combined with subjective reports on cognitive decline. Dementia diagnosis was based on all available information including medical files, neuropsychological testing, temporal cognitive decline detected either by the repeated neuropsychological evaluations, interview by the study nurse to detect functional decline or information from family members.

### Blood samples

Venous blood was collected on 758 occasions, where over 80% were collected between baseline and the three year follow-up, with a mean and median of 3.0 blood samples per participant during the period.

### Telomere length PCR

Genomic DNA was extracted from buffy coat with the MagAttract DNA Blood Midi kit (Qiagen) in the BioRobot M48 Workstation and DNA purity and concentration measured by the Nanodrop instrument (Thermo scientific). Telomere length was determined by the quantitative-PCR method described by Cawthon [20]. Briefly, DNA was diluted to 2.19 ng/ul in TE/E.coli buffer and Telomere (Tel) and single copy gene (HBG) reactions were run separately in a 384 well optical plate (MicroAmp, Applied biosystems) in triplicates on the ABI7900HT instrument (Applied Biosystems). Each reaction contained: 17.5 ng DNA, 0.1 µM forward Tel primer/0.4 µM forward HBG primer, 0.9 µM reverse Tel primer/0.4 µM reverse HBG, 1X PCR Buffer 2, 1.7 mM (Tel)/2.5 mM (HBG) MgCl2, 2.5 mM (Tel)/5 mM (HBG) DTT, 0.2 mM dNTP, 150 nM ROX, 0.2X SYBR and 0.625 U AmpliTaq Gold (Applied Biosystems).

Telomere and HBG primer sequence written 5′–3′ were: Tel forward: CGGTTTGTTTGGGTTTGGGTTTGGGTTTGGGTTTGGGTT, Tel reverse: GGCTTGCCTTACCCTTACCCTTACCCTTACCCTTACCCT, HBG forward: TGTGCTGGCCCATCACTTTG and HBG reverse: ACCAGCCACCACTTTCTGATAGG.

Telomere (Tel)/single copy gene (HBG) (T/S) values were calculated by 2^−ΔCt^ method, where ΔCt =  average Ct_Tel_−average Ct_HBG_. Relative telomere length (RTL) were generated by dividing samples T/S value with the T/S value of a reference cell line DNA (CCRF) included in all runs. A standard curve generated by the reference DNA was also included in each run to monitor PCR efficiency. The mean interassay coefficient of variation regarding relative telomere length (RTL) for this method was 7%.

### Statistical analyses

Statistical analyses were performed with IBM SPSS Statistics 21 and the Tableau software was used for selected visualizations. Statistical significance of differences in characteristics of patient and control groups at baseline was assessed using the median test for independent samples. Pairwise test (Mann–Whitney) was used to compare median age in individual patient groups and control. Distribution of gender and cognitive state at baseline between the patient and control groups were compared using Pearson chi-square test. Analyzes of RTL were performed using ANCOVA (analysis of covariance) with age as a covariate, no significant interaction between age and any group tested was found (homogeneity of regression slopes). P-values for the comparison of patient groups to controls were obtained through post hoc tests without adjustments for multiple comparisons. Mean telomere attrition rate over a three year period in PD patients and in controls was tested using paired t-tests. Analysis was restricted to individuals with RTL data at baseline and three year follow up. The significance of the differences between men and women regarding disease progression was obtained through Pearsons chi-squared test.

The RTL for the PD patient group was divided into two groups, short and long, where long, are the RTLs higher than the median value of 0.75 and short, are RTLs smaller or equal to the median. Survival analysis of developing dementia (within three years) was performed on the RTL groups. The Kaplan Meier estimator was used to estimate the cumulative survival (no dementia) function and significance was obtained through Log-rank test (Mantel Cox). The hazard ratios between RTL groups long/short were obtained using Cox regression with age as covariate.

To estimate the RTL variation across time the standard deviations and coefficient of variations (CV) across follow ups (up to five years) were calculated for each individual. Differences in mean standard deviations and mean CV between patient and control groups were tested using ANOVA (analysis of variance).

## Results

### Baseline characteristics

The patient cohort with idiopatic parkinsonism consisted of 136 patients diagnosed with PD, 17 with PSP, 15 with MSA, and 30 healthy controls. Blood samples were collected at baseline and at yearly follow up for up to five to seven years. Baseline characteristics of patients and controls are described in Table 1. The median age where similar in PD and control group but PSP and MSA patients were significantly older (p<0.05) than the control group. The distribution of men and women was similar in all groups.

**Table 1:** Baseline characteristics of 168 patients with idiopathic parkinsonism and 30 controls.

Baseline characteristics			Controls	PD	PSP	MSA
**Participants at baseline**		*n*	30	136	17	15
**Age baseline (year)**		*median (range)*	69.6 (48–78)	71.4 (40–90)	74.9* (60–86)	75.6* (46–88)
**Disease duration at baseline (month)**		*median (range)*	-	16.5 (2.5–176.5)	16.8 (3.5–61.6)	19.6 (2.7–36.4)
**Sex**		*Male (n)*	16	80	9	10
		*Female (n)*	14	56	8	5
**Hoehn Yahr stage**		*Median (range)*	-	2 (1–5)	2.5 (1.5–5)	2.5 (2–5)
**UPDRS - Total**		*Median (range)*	-	35 (8–81)	34 (20–81)	39 (12–76)
**UPDRS - III Subtotal**		*Median (range)*	-	26 (5–62)	26 (12–64)	27 (9–48)
**Cognitive state baseline**	**Normal**	*n*	26	80	5	11
	**MCI**	*n*	4	56*	12*	4*
	**Dementia**	*n*	0	0	0	0

*  = p<0.05 vs control, n =  number, UPDRS: Unified Parkinson's Disease Rating Scale, PD: Parkinson's disease, PSP: Progressive supranuclear palsy, MSA: Multiple system atrophy, MCI: Mild cognitive impairment.

The Hoehn and Yahr stages and the UPDRS scores (total and subtotal motor section III) median subscores did not differ significantly between the patient groups. The patient groups showed a higher proportion of individuals with mild cognitive impairment (MCI) compared to control at baseline (p<0.05). No individual had dementia at diagnosis.

**Figure 1:**
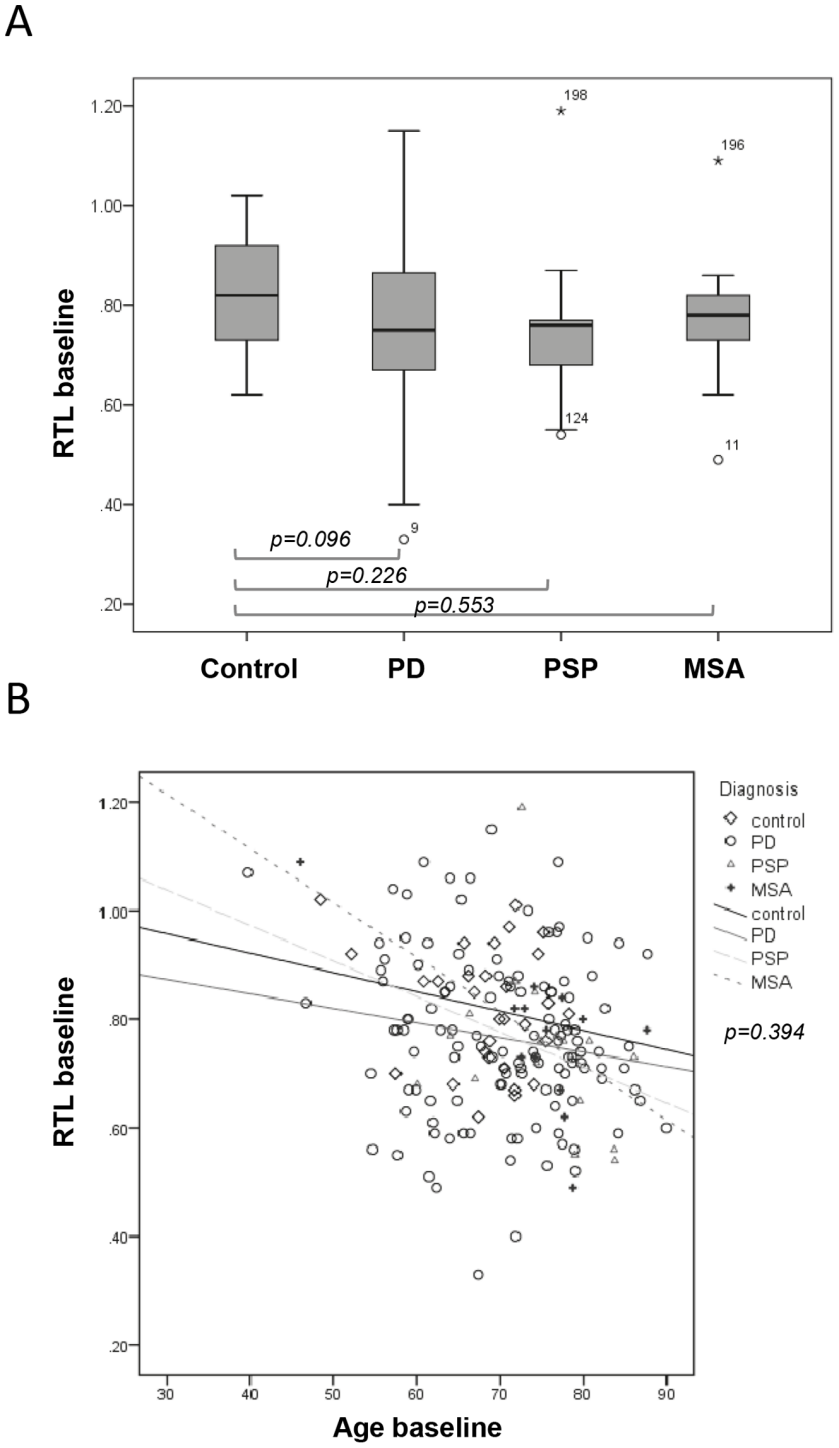
Telomere length characteristics at baseline. A) Mean relative telomere length (RTL) at baseline in control (n = 30), PD (n = 124), PSP (n = 17) and MSA (n = 13) samples. Age-adjusted p-values comparing control with patient subgroups. B) RTL as a function of age at baseline with regression lines. Correlation between telomere length and age in control and patient subgroups examined using the Pearson's correlation coefficient. No significant (p = 0.394) difference in the relationship between RTL and age was found in control and patient groups.

The mean RTL at baseline was 0.82 in controls compared to 0.77 in PD, 0.74 in PSP and 0.77 in MSA (Fig. 1A and Table 2). Although there was a tendency of shorter mean RTL in the patient groups it was not significant after age adjustment (Fig. 1A). Telomere length correlated with age at diagnosis in both control and patients, with shorter RTL at higher age. The negative correlation between telomere length and age at baseline was similar in all groups (p = 0.394) (Fig. 1B).

**Table 2:** Mean RTL at diagnosis and up to five year follow up.

Mean RTL (StDev)	Control	PD
**Baseline**	0.82 (0.11)	0.77 (0.15)
**n**	30	124
**One year**	-	0.74 (0.14)
**n**	0	124
**Two years**	-	0.72 (0.14)
**n**	0	114
**Three years**	0.76* (0.10)	0.71* (0.14)
**n**	21	88
**Four years**	-	0.70 (0.14)
**n**	0	62
**Five years**	0.75* (0.12)	0.71* (0.14)
**n**	18	47

Mean RTL did not differ significantly between males and females in any of the groups (data not shown).

### Telomere length dynamics

Blood was collected at baseline (diagnosis) and at yearly follow up in PD subgroups up to seven years and at baseline and three/five years follow up in controls (S1 Table). The mean RTL decreased by time and the shortening between baseline and three/five years follow up was significant in both control (p<0.05) and PD (p<0.05), whereas the number of individuals followed in MSA and PSP were too few for detailed analysis (Table 2). The attrition rate at three/five years was similar in PD and control groups (Table 2 and data not shown).

**Figure 2:**
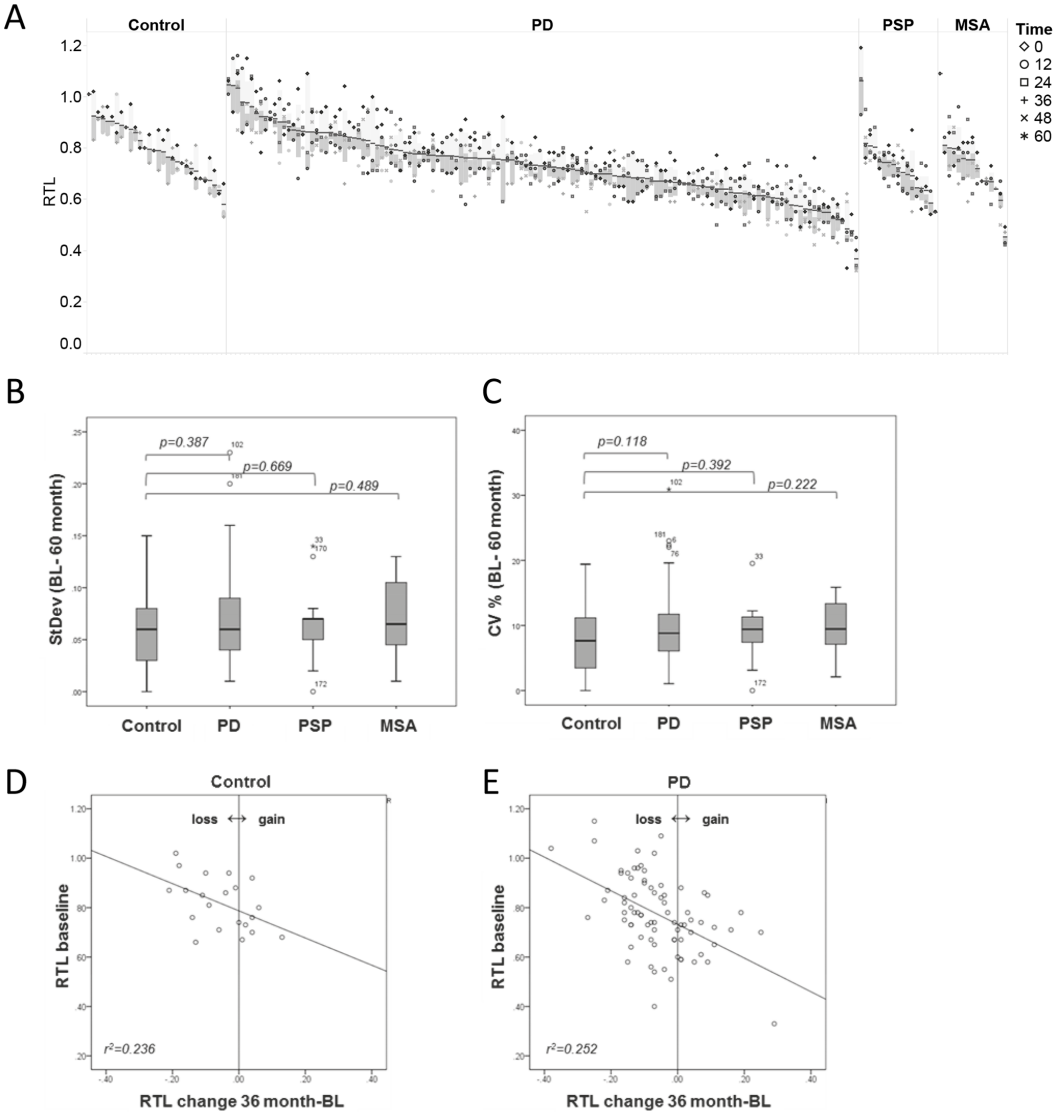
Telomere length dynamics. A) Individual relative telomere length (RTL) at diagnosis and at repeated follow up for up to five years in control, PD, PSP and MSA groups. RTL quartiles marked in each individual. B–C) Std.Dev (B) and CV% (C) of RTL in control, PD, PSP and MSA groups from baseline and up to five years follow up. D–E) Individuals RTL change between baseline and three year follow up in control (D) and PD (E).

The overview of telomere length dynamics in control, PD, PSP and MSA individuals at baseline and up to five years follow up showed a wide span in RTL between individuals (Fig. 2A). However, individual's telomere length seems rather stable over time in all groups (Fig. 2A). Telomere length variation over time was tested and no significant differences in std.dev or CV (%) (baseline and up to five year follow up) was observed in the control, PD, PSP or MSA groups (Fig. 2B–C).

Individuals change in RTL over a three year period in control and PD patients show similar tendency, individuals with longer telomeres at baseline are more likely to shorten their telomeres than patients with shorter telomeres (Fig. 2 D–E).

### RTL and disease progression

**Figure 3:**
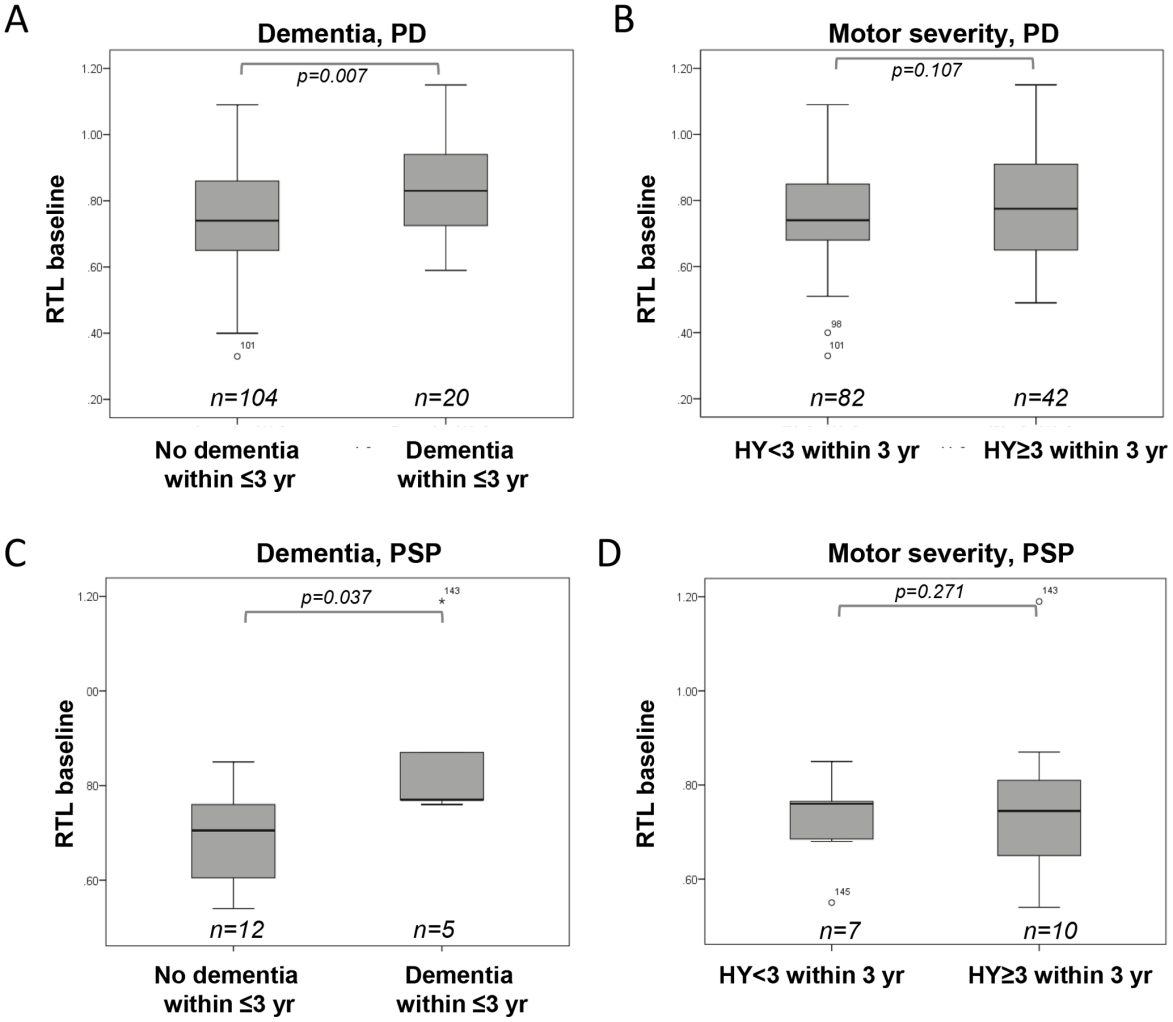
RTL and disease progression. Relative telomere length (RTL) at diagnosis was analyzed in relation to dementia progression and motor severity stage within three years from diagnosis. A, C) Mean RTL was significantly longer in A) PD (p = 0.007) and C) PSP (p = 0.037) patients who developed dementia within three years from diagnosis. B, D) Mean RTL in patients by motor severity defined as Hoehn and Yahr stage <3 or stage 3 or higher did not differ in B) PD (p = 0.107) or D) PSP (p = 0.271) groups.

**Figure 4:**
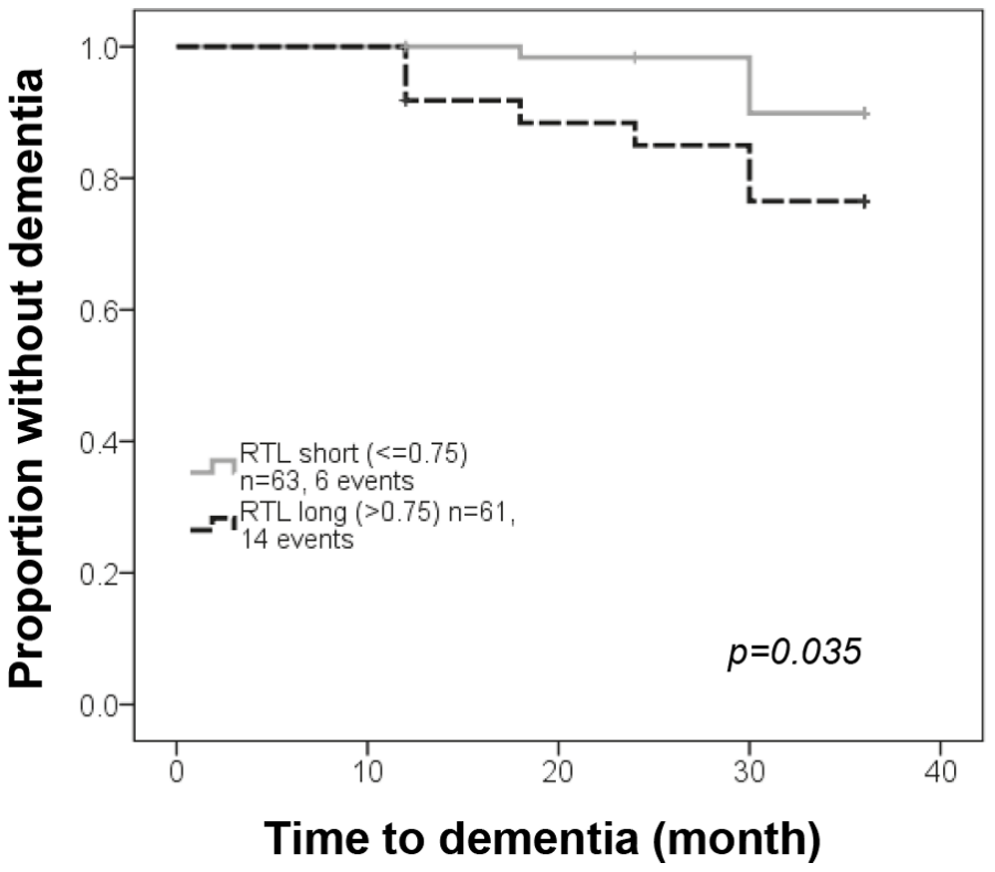
Dementia progression in PD patients with long and short telomeres at diagnosis. Proportion of patients without dementia with long (RTL>0.75) vs. short (RTL≤0.75) leukocyte telomeres using Kaplan-Meier with the log-rank test.

Disease progression monitored as cognitive and motor decline was analyzed in relationship to RTL at diagnosis (S1 Table). In PD and PSP the mean RTL was significantly longer (p = 0.007 and p = 0.037 respectively) in patients who developed dementia within three years from baseline (Fig. 3 A, C). No patient with MSA developed dementia. In contrast, no difference in mean RTL was seen (p = 0.107 (PD), p = 0.271 (PSP), p = 0.397 (MSA)) between patients who during the first three years developed a more sever motor stage (defined as HY stage three or higher) vs those who did not (HY<3) (Fig. 3 B, D and data not shown). Furthermore, RTL at baseline was not related to UPDRS scores (total and motor part III) at three year follow up in any patient group (data not shown). In PD, no difference between men and women regarding dementia progression (p = 0.358, Chi square) was observed. Event free Kaplan Meier survival analysis with progression into dementia defined as event was done, where the PD patients was separated into two RTL groups (short RTL< = 0.75, long RTL>0.75) based on the median RTL value. It showed a significant higher proportion of patients with progression to dementia within three years follow up in patients with long telomeres at baseline (p = 0.035) (Fig. 4).

In PD, age-adjusted HR estimation based on RTL groups (short/long telomeres) at diagnosis showed a significant (p = 0.01) increased risk of developing dementia within three or five years (HR 3.7 and 2.7 respectively). On the contrary, telomere length at baseline was not related to mortality (Table 3).

**Table 3:** Telomere length (RTL) at baseline and risk (HR) of disease progression in PD.

Long (< = 0.75) vs. short (>0.75) RTL in PD	HR	p-value
Dementia progression within three year from baseline	3.68	0.01
Dementia progression within five year from baseline	2.71	0.01
Death during FU	1.07	0.88

*Age  =  covariate.*

## Discussion

In our community-based study population with idiopathic parkinsonism we have analyzed leukocyte telomere length in relation to disease status at diagnosis and disease progression during a three to five year follow up period.

At diagnosis the mean RTL did not differ significantly between the control and PD, PSP or MSA group. This is in line with studies from England and Finland which found no difference in their PD samples and controls [8], [11]. PSP and MSA are disorders with early affection of both pre- and postsynaptic dopaminergic neurons and a more rapid disease progression and shorter time to death compared to PD. Thus, the more devastating disease processes in PSP and MSA compared to PD could envisage telomeres being more affected in PSP and MSA but our results do not show any differences between the three disorders, neither at baseline nor longitudinally.

As expected we found a negative relationship between age and RTL at diagnosis, in line with what most others [8]–[10], [21] but not all [7], [11] PD-studies have found. The negative relationship between telomeres and age was also seen in the control group and regarded as a general non-disease related phenomenon. Telomere shortening over a three year period was similar in PD and control groups and most apparent in individuals with long telomeres at baseline. This phenomena has been described previously in a number of publications including cancer associated studies [22].

PD is a heterogeneous disorder with occurrence of different motor, cognitive and other phenotypes, seen already in early stages. Markers for improved characterization of these phenotypes are desired to improve understanding of disease mechanisms and to improve care. The underlying cellular dysfunction in subgroups with PD most severely affected in motor and cognitive functions may be viewed as effects of cellular dysfunction due to mechanisms related to age (genetic and cumulative environmental life time exposures). Based on the reported effects of genetic and environmental factors on telomere length homeostasis [2] we analyzed RTL in relation to phenotype. A significant association between longer telomere length at diagnosis and increased risk of developing dementia within three years from diagnosis was observed in both PD and PSP. In contrast, we found no association between motor phenotype or mortality and RTL in any of the PD, PSP or MSA groups.

To our knowledge this is the first study showing a prognostic relevance of RTL at diagnosis with regard to dementia progression. The mechanism behind the connection of long RTL in leukocytes and dementia is not known. We can only speculate and based on our previous studies on renal cell carcinoma patients we have shown the potential influence of cytokines and T-regulatory cells (Treg) fractions on blood telomere length [23]. Treg cells are known to suppress immune responses and the observation that patients with higher Treg levels had longer blood telomeres could reflect a suppressed immune system with fewer cell divisions and therefore less telomere shortening. There are a number of publications indicating a neuroprotective function of Tregs in Parkinson's disease [24]–[26]. Unfortunately we did not have any detailed data on the fraction of Treg cells in the patients and controls. Future studies will focus on combining immunological data with oxidative stress markers in relation to telomere length in different blood cell fractions.

Moreover, it would be of interest to expand the analysis to study if regional telomere length differences exists in brain tissue in patients and controls and in relation to dementia progression. So far only few studies have measured telomere length post-mortem in brain tissue and in a small study of Parkinson's disease no difference was found in substantia nigra telomere length between patients and controls [11]. Of special interest would be to relate blood and brain tissue telomere length to protein folding or degradation related to synuclein aggregation in brain tissue [27]. These types of studies will further evaluate the reasons behind the increased risk for patients with long leukocyte telomeres at baseline in developing dementia.

A limitation of the present study was the relatively small number of controls. However for most of the controls we were able to collect blood longitudinally, for up to five years. The number of patients with PSP and MSA, which are rare disorders that are less common than PD, was also small and therefore the results for PSP and MSA should be interpreted cautiously. Another limitation is the lack of histo-pathologic verification of the diagnosis of PD, PSP and MSA. It is however likely that the diagnoses are correct as diagnoses were made independently by two movement disorder specialists and were reevaluated yearly and that established diagnostic criteria were used. All patients had abnormal uptake on SPECT FP-CIT scans showing abnormal presynaptic function as found in PD and atypical forms of parkinsonism and all controls had normal uptake indicating normal function and no preclinical parkinsonism.

The strengths of the study were that it was population based with nearly complete case-finding in the catchment area which makes it possible to generalize the results; a prospective clinical follow-up with rigorous application of diagnostic criteria and a follow up time of at least four years; investigation of RTL in patient populations not investigated before (longitudinal in PD and cross-sectional and longitudinal in PSP and MSA) and exploring a possible association between RTL and phenotypes.

## Conclusions

To conclude, this is the first study of RTL in a patient population with different forms of idiopathic parkinsonism, and with longitudinal blood sampling. RTL did not differ between control and patient groups at diagnosis and no difference in telomere stability or attrition was found between PD patients and controls. Telomere length at diagnosis could not be associated with motor severity but interestingly PD and PSP patients with the longest telomeres at diagnosis had increased risk of developing dementia within three years follow up. The mechanism behind the connection of long RTL in leukocytes and dementia progression is not known and needs further investigation.

## Supporting Information

S1 Table RTL, Hoehn and Yahr stage and cognitive status at baseline and up to five years follow up.
(PDF)
