# Supplementary material for: Long Leukocyte Telomere Length at Diagnosis Is a Risk Factor for Dementia Progression in Idiopathic Parkinsonism
Source: PLoS One. 2014 Dec 12;9(12):e113387. doi: 10.1371/journal.pone.0113387 (PMC4264694; doi:10.1371/journal.pone.0113387)
Supplement: S1 Table — RTL, Hoehn and Yahr stage and cognitive status at baseline and up to five years follow up. (PDF) [file pone.0113387.s001.pdf]

Supplementary Table S1

Patient	*Diagnosis*	*Age at baseline*	*RTL baseline*	*RTL 12 month FU*	*RTL 24 month FU*	*RTL 36 month FU*	*RTL 48 month FU*	*RTL 60 month FU*	*Hoehn and Yahr stage baseline*	*Hoehn and Yahr stage 12 month FU*	*Hoehn and Yahr stage 36 month FU*	*Hoehn and Yahr stage 60 month FU*	*HY stage ≥ 3 within 3 yr from baseline*	*Cognitive status baseline*	*Cogn_stat_ 12 month FU*	*Cogn_stat_ 24 month FU*	*Cogn_stat_3 6 month FU*	*Cogn_stat_ 48 month FU*	*Cogn_stat_ 60 month FU*	*Dementia progression within ≤ 3 years*
8	PD	64	1.06	0.99	0.82			0.74	3	2.5	3	2.5	Yes	Normal	Normal	Normal	Normal	Normal	MCI	No
9	PD	56	0.89	0.84		0.84	0.76	0.71	2	2	2	2	No	Normal	Normal	Normal	Normal	Normal	Normal	No
10	PD	81		0.74	0.77	0.68	0.72	0.82	1	2.5	3	3	Yes	Normal	Normal	Normal	Normal	Normal	MCI	No
18	PD	61	1.09	1.16	1.10	1.04	0.87	0.94	1	1	1.5	2	No	Normal	Normal	Normal	Normal	Normal	Normal	No
19	PD	77	0.76	0.63	0.57	0.49	0.42	0.47	1	1	2	2	No	Normal	Normal	Normal	Normal	Normal	Normal	No
20	PD	70	0.68	0.65	0.68	0.57	0.59	0.57	2	2.5	2	2	No	Normal	Normal	Normal	MCI	Normal	Normal	No
23	PD	71	0.54	0.47	0.46	0.47		0.44	1	1	2	2	No	Normal	MCI	MCI	MCI	MCI	MCI	No
24	PD	84	0.94	1.15					4	4	5		Yes	MCI	MCI	Dementia	Dementia			Yes
33	PD	55	0.94	0.82	0.95	0.77	0.90	0.79	2	2	2	2	No	Normal	Normal	Normal	Normal	Normal	Normal	No
34	PD	63	0.85	0.71	0.80	0.81	0.83	0.63	2	2	2	2	No	Normal	Normal	Normal	Normal	Normal	Normal	No
37	PD	65	0.73	0.78		0.74		0.86	2	2	2	2	No	Normal	Normal	Normal	Normal	Normal	Normal	No
39	PD	77		0.62	0.75	0.66	0.52	0.50	2	3	2	4	Yes	Normal	Normal	Normal	Normal	Normal	MCI	No
42	PD	55		0.87	0.89		0.86	0.73	2	2	2	4	No	MCI	MCI	MCI	MCI	MCI	Dementia	No
43	PD	83	0.82		0.78	0.66			3	3	5		Yes	MCI	MCI	MCI	Dementia			Yes
46	PD	55		0.76	0.75	0.66	0.83	0.77	2	2	3	3	Yes	Normal	Normal	Normal	Normal	Normal	Normal	No
53	PD	65	0.75	0.68	0.62				2	2	2	2	No	Normal	Normal	Normal	Normal	Normal	Normal	No
57	PD	62	0.82	0.74	0.84	0.78	0.73	0.77	2.5	2	2	2	No	MCI	MCI	MCI	MCI	MCI	MCI	No
59	PD	63		0.55	0.61	0.55	0.55	0.61		1	2	2	No	Normal	MCI	MCI	MCI	Normal	Normal	No
62	PD	69	0.84	0.72	0.81	0.79	0.72	0.69	3	1	2.5	2.5	Yes	Normal	Normal	Normal	Normal	Normal	Normal	No
64	PD	77	0.64	0.52	0.67	0.50	0.57	0.56	3	3	3	4	Yes	MCI	Normal	Normal	Normal	Normal	Normal	No
65	PD	82	0.74	0.67	0.72	0.66		0.71	2	2	2	2	No	Normal	Normal	Normal	Normal	Normal	Normal	No
67	PD	56	0.87	0.97	0.90			0.79	2	2		2	No	MCI	Normal	Normal	Normal	Normal	MCI	No
68	PD	70			0.76	0.77	0.71	0.79			2.5	4	No	MCI	MCI	MCI	MCI	MCI	MCI	No
72	PD	62	0.65	0.69	0.60	0.76	0.71	0.72	3	3	2	2	Yes	MCI	MCI	MCI	MCI	MCI	MCI	No
73	PD	64	0.86	0.87	0.83	0.94	0.81	0.85	2	3	2	2.5	Yes	MCI	MCI	MCI	MCI	MCI	MCI	No
74	PD	65	0.65	0.72	0.67	0.58	0.66	0.62	2	2	2	2	No	MCI	MCI	MCI	MCI	MCI	MCI	No
78	PD	58	0.55	0.61	0.66	0.51	0.52	0.52	2	2	2	2	No	Normal	Normal	Normal	Normal	Normal	Normal	No
79	PD	72	0.85	0.78	0.68	0.72	0.66	0.86	1.5	2	2	3	No	MCI	MCI	MCI	Dementia	Dementia	Dementia	Yes
81	PD	57	0.78	0.74	0.94	0.81	0.84	0.67	1	1	1	2	No	Normal	Normal	Normal	Normal	Normal	Normal	No
86	PD	40	1.07		1.08	0.82	0.87	0.96	1		2	2	No	Normal	Normal	Normal	Normal	Normal	Normal	No
87	PD	78	0.73	0.65	0.58	0.59	0.51	0.56	2.5	2	2.5	4	No	Normal	Normal	Normal	Normal	Normal	Normal	No
89	PD	76	0.67	0.78	0.63	0.59			2	2	3		Yes	MCI	MCI	Dementia	Dementia			Yes
96	PD	59	0.95		0.80	0.78	0.71	0.77	2.5		2	2	No	Normal	Normal	Normal	Normal	Normal	MCI	No
97	PD	58	0.78	1.03	0.98	0.97	0.86	0.92	1	1.5	2	2	No	Normal	Normal	Normal	Normal	Normal	Normal	No
101	PD	73	0.70	0.94	0.81	0.95	0.80	0.77	2	2.5	3	3	Yes	MCI	MCI	MCI	MCI	Dementia	Dementia	No
106	PD	76	0.85	0.95	0.88		0.92	0.91	2	2	4	5	Yes	Normal	Normal	Normal	MCI	Dementia	Dementia	No
114	PD	77	0.96	0.76	0.70	0.83	0.91		3	5	4		Yes	MCI	Dementia	Dementia	Dementia	Dementia	Dementia	Yes
118	PD	70	0.74	0.75	0.76	0.67	0.65	0.70	2	2	2	2	No	MCI	Normal	Normal	Normal	Normal	Normal	No
119	PD	83			0.68	0.67	0.66		5	5	4	4	Yes	MCI	MCI	Dementia	Dementia	Dementia	Dementia	Yes
133	PD	62	0.49	0.69			0.48	0.63	2	2.5	3	2	Yes	Normal	Normal	Normal	Normal	Normal	Normal	No
141	PD	65	1.02	0.94		0.95	0.86	0.91	1.5	1.5	2.5	2.5	No	Normal	MCI	MCI	MCI	Dementia	Dementia	No
146	PD	67	0.77	0.73	0.61	0.66	0.76	0.77	2	2	2	2	No	MCI	MCI	MCI	MCI	Dementia	Dementia	No
150	PD	67	0.33	0.47	0.44	0.62	0.59	0.45	2.5	2	2	2	No	MCI	MCI	MCI	MCI	MCI	MCI	No
151	PD	60	0.67	0.67	0.60	0.66	0.63	0.70	2	2	2	2	No	Normal	Normal	Normal	Normal	Normal	Normal	No
157	PD	79	0.73	0.68	0.50	0.59	0.54	0.69	2.5	2.5	2	2	No	Normal	Normal	Normal	Normal	Normal	Normal	No
171	PD	79	0.78	0.73	0.74	0.65			4	5	4	4	Yes	Normal	Normal	Normal	MCI	Dementia	Dementia	No
173	PD	47	0.83	0.86	0.70	0.61	0.83	0.74	2	2	2	2	No	Normal	Normal	Normal	MCI	Normal	Normal	No
174	PD	77		0.60	0.63	0.61			2.5	2.5	3		Yes	MCI	MCI	Dementia	Dementia	Dementia	Dementia	Yes
175	PD	80	0.95	0.80	0.86	0.85		0.79	3	2	2	2.5	Yes	MCI	MCI	MCI	MCI	Dementia	Dementia	No
177	PD	61	0.51	0.58	0.59	0.49	0.55	0.64	2.5	2	2	2	No	Normal	Normal	Normal	Normal	Normal	Normal	No
180	PD	63	0.78	0.73	0.73	0.66	0.71	0.58	2	2	2.5	2	No	Normal	Normal	Normal	Normal	Normal	MCI	No
187	PD	70	0.88	0.95	0.71	0.80	0.73	0.80	2	2.5	2	2	No	Normal	Normal	Normal	Normal	Normal	Normal	No
188	PD	84	0.59	0.73	0.52	0.60	0.66	0.46	3	3	3	2.5	Yes	Normal	Normal	Normal	Normal	Normal	MCI	No
195	PD	71	0.87	0.81	0.61	0.66	0.87		2.5	1	2	2	No	Normal	Normal	Normal	Normal	Normal	Normal	No
196	PD	59	0.80	0.70		0.66	0.64		2	1.5	2	2	No	Normal	Normal	Normal	Normal	Normal	Normal	No
198	PD	71	0.58	0.53	0.54	0.43	0.57	0.48	2.5	1	2	2	No	MCI	Normal	Normal	Normal	Normal	MCI	No
199	PD	76	0.85		0.88	0.94	0.86	0.80	2.5	2.5	2	2.5	No	MCI	MCI	Dementia	Dementia	Dementia	Dementia	Yes
206	PD	74	0.74	0.72	0.66	0.81			2.5	2	2	2	No	MCI	MCI	MCI	MCI	MCI	MCI	No
208	PD	79	0.65	0.55	0.64				3	3	2.5		Yes	MCI	MCI					No
211	PD	76		0.62	0.74	0.51	0.61		2	3	3		Yes	MCI	MCI	MCI	MCI	MCI		No
213	PD	88	0.92		0.59				5	4			Yes	Normal	Normal					No
216	PD	65	0.92	0.81	0.85	0.78	0.90		1.5	3	2		Yes	Normal	Normal	Normal	Normal	Normal	Normal	No
218	PD	69	1.15	0.89		0.90	0.97		3	2	4	4	Yes	Normal	MCI	MCI	Dementia	Dementia	Dementia	Yes

219	PD	80	0.71		0.61	0.64			3	3	4		Yes	Normal	Normal	Normal	Normal			No
239	PD	57	1.04	0.85		0.66	0.83		2	2	1.5		No	Normal	Normal	Normal	Normal	Normal	Normal	No
240	PD	74	0.73	0.65	0.71	0.64			2	2	3		Yes	MCI	MCI	MCI	Dementia	Dementia	Dementia	Yes
245	PD	61	0.94	0.94	0.84	0.79	0.79		2.5	2	2	2	No	MCI	Dementia	Dementia	Dementia	Dementia	Dementia	Yes
257	PD	69	0.73	0.77		0.75	0.69		1	2	2	2	No	MCI	Normal	Normal	Normal	Normal	Normal	No
265	PD	72	0.40	0.45	0.34	0.33	0.32		2.5	2	2	2	No	MCI	Normal	Normal	Normal	Normal	Normal	No
274	PD	55	0.56	0.64	0.57	0.48	0.52		2	2	3	3	Yes	Normal	Normal	Normal	Normal	Normal	MCI	No
278	PD	71	0.86	0.67	0.88	0.79	0.69		3	2	2	2	Yes	Normal	Normal	Normal	Normal	Normal	MCI	No
282	PD	81	0.88	0.80					2.5	2.5			No	Normal	Normal	Normal	Normal			No
284	PD	77	0.71	0.91	0.71	0.87	0.75		2	2	2	2	No	Normal	Normal	Normal	Normal	Normal	MCI	No
288	PD	82	0.71	0.71	0.61	0.71	0.61		3	4	5		Yes	MCI	MCI	MCI	Dementia	Dementia	Dementia	Yes
291	PD	79	0.52	0.52	0.51				3	4	4		Yes	MCI	MCI	MCI	MCI	Dementia	Dementia	No
294	PD	70	0.91	0.58		0.81	0.75		3	2	2	2	Yes	MCI	MCI	MCI	MCI	MCI	MCI	No
295	PD	71	0.70	0.78	0.75	0.74	0.55		2	2	2	2	No	Normal	Normal	Normal	Normal	Normal	Normal	No
298	PD	44		0.81	0.73	0.89	0.74		2	2	2	2	No	Normal	Normal	Normal	Normal	Normal	Normal	No
301	PD	68	0.75	0.61	0.65	0.79	0.66		2	2	2	2	No	MCI	MCI	MCI	MCI	MCI	Dementia	No
309	PD	76	0.96	0.78		0.84			2	2	2	3	No	Normal	Normal	Normal	MCI	MCI	MCI	No
311	PD	72	0.58	0.51	0.57	0.63	0.46		2	2	2	2	No	Normal	Normal	MCI	MCI	MCI	MCI	No
312	PD	82	0.69	0.73	0.68				2.5	2.5			No	Normal	Normal	Normal	Normal			No
315	PD	64	0.78	0.72	0.80	0.62	0.68		2	2	2	2	No	MCI	MCI	MCI	MCI	MCI	MCI	No
320	PD	58	0.78	0.71	0.66	0.75			2	2	2	2	No	Normal	Normal	Normal	Normal	Normal	Normal	No
321	PD	90	0.60	0.58	0.62				4	4			Yes	Normal	Normal					No
323	PD	78	0.84	0.72	0.69	0.68			3	3	4	5	Yes	MCI	MCI	MCI	Dementia	Dementia	Dementia	Yes
324	PD	80	0.74	0.61	0.60	0.73			2	2	2	2	No	Normal	Normal	Normal	MCI	MCI	MCI	No
325	PD	77	0.57	0.57	0.51				3	2			Yes	Normal	Normal	Dementia				No
328	PD	74	0.77	0.63	0.50	0.66			2	2	3		Yes	MCI	MCI	MCI	Dementia	Dementia	Dementia	Yes
332	PD	79	0.72		0.75	0.83			2.5	1.5	2	2.5	No	Normal	Normal	Normal	Normal	Normal	Normal	No
335	PD	72	0.88	0.82	0.85	0.89			2.5	2	2	2	No	Normal	Normal	Normal	Normal	Normal	Normal	No
336	PD	59	0.67	0.77	0.77	0.68			1.5	2	2.5	2	No	Normal	Normal	Normal	MCI	MCI	MCI	No
338	PD	77			0.66				3	2.5	3	3	Yes	Normal	Normal	Normal	MCI	Dementia	Dementia	No
340	PD	64		0.79		0.67			1.5	1.5	2	2	No	MCI	MCI	MCI	Normal	MCI	MCI	No
355	PD	59	1.03	0.68	0.85	0.91			2	2	2		No	MCI	MCI	MCI	Dementia	Dementia	Dementia	Yes
356	PD	74	0.60	0.61	0.65	0.60			3	2	2	2	Yes	Normal	Normal	Normal	Normal	Normal	MCI	No
367	PD	77	0.67	0.59		0.66			2	2	2.5		No	Normal	MCI	MCI	MCI	MCI	Dementia	No
371	PD	62	0.61	0.68	0.62	0.68			2	2	2	2	No	Normal	MCI	MCI	MCI	MCI	MCI	No
380	PD	86	0.75	0.64	0.68	0.59			3	3	3		Yes	MCI	Normal	Normal	Normal	Normal		No
383	PD	85	0.71	0.72	0.70				2	2	3	4	Yes	MCI	MCI	MCI	MCI	MCI	Dementia	No
384	PD	77	0.59	0.64	0.59	0.60			2	2.5	2	2	No	MCI	MCI	MCI	Dementia	Dementia	Dementia	Yes
388	PD	60	0.90		0.83	0.80			1.5	1.5	2	2	No	Normal	Normal	Normal	Normal	Normal	Normal	No
389	PD	64	0.58	0.64	0.70	0.67			2	2	2		No	Normal	Normal	Normal	Normal	Normal	Normal	No
393	PD	77	0.97	0.93	0.90	0.86			3	4	5		Yes	MCI	MCI	Dementia	Dementia			Yes
402	PD	87	0.65	0.73					3	3	5		Yes	Normal	MCI	MCI	MCI			No
403	PD	86	0.67	0.66					1.5	3			Yes	MCI	MCI	MCI				No
405	PD	70	0.68	0.71	0.71				2	2	2	2	No	MCI	Normal	Normal	Normal	Normal	MCI	No
410	PD	59	0.80	0.75	0.78				1.5	2	2	2	No	Normal	Normal	Normal	Normal	Normal	Normal	No
412	PD	78	0.70	0.62	0.66				2	2	2	2.5	No	Normal	Normal	Normal	MCI	Dementia	Dementia	No
413	PD	66	0.59	0.61	0.67				2	2	2	2	No	Normal	Normal	Normal	Normal	Normal	Normal	No
414	PD	63	0.85	0.70	0.76				2	2	2	2	No	Normal	MCI	MCI	MCI	MCI	MCI	No
415	PD	73	0.71	0.67	0.63				2	2	2		No	MCI	MCI	MCI	Dementia	Dementia	Dementia	Yes
422	PD	78	0.87	0.99	0.91				3	2.5	2.5	3	Yes	Normal	Normal	Normal	Normal	Normal	Normal	No
426	PD	62	0.59	0.70	0.77				1.5	2	2	2	No	Normal	Normal	Normal	Normal	Normal	Normal	No
431	PD	72	0.72	0.72	0.84				2.5	2	2	2	No	MCI	MCI	MCI	Dementia	Dementia	Dementia	Yes
432	PD	66	0.89	0.83	0.89				2	2	2	2	No	Normal	Normal	Normal	MCI	Dementia	Dementia	No
436	PD	77	0.78	0.79	0.75				3	3	3		Yes	MCI	Dementia	Dementia	Dementia	Dementia	Dementia	Yes
441	PD	73	1.00	0.75	0.71				2	2	2	2	No	Normal	Normal	Normal	Normal	Normal	Normal	No
443	PD	78	0.79	0.75					2	4			No	MCI	Dementia	Dementia	Dementia	Dementia	Dementia	Yes
445	PD	59	0.63	0.67	0.75				2	2	2	2	No	Normal	Normal	Normal	Normal	Normal	Normal	No
446	PD	77	1.09	0.72	0.78				2	2	2	4	No	MCI	MCI	MCI	MCI	Dementia	Dementia	No
453	PD	77	0.77	0.82	0.76				2	2	2	2	No	Normal	Normal	Normal	Normal	Normal	MCI	No
454	PD	79	0.56	0.68	0.64				2	2	2	3	No	Normal	Normal	Normal	MCI	MCI	MCI	No
455	PD	79	0.76		0.74				3		4		Yes	Normal	MCI	MCI	MCI	MCI	MCI	No
458	PD	66	1.06	1.01	1.07				3	2	2	2	Yes	Normal	Normal	Normal	Normal	Normal	Normal	No
460	PD	56	0.91	0.94	0.91				3	2	2	2	Yes	Normal	Normal	Normal	MCI	MCI	MCI	No
464	PD	76	0.53	0.60	0.45				2	2	2		No	MCI	MCI	MCI	MCI	Dementia	Dementia	No
467	PD	78	0.78	0.82	0.71				2	2	2	2	No	MCI	MCI	MCI	MCI	MCI	Normal	No
473	PD	80	0.72	0.81	0.74				2	2	2	2	No	Normal	Normal	Normal	MCI	MCI		No
479	PD	76	0.80	0.72					3	3			Yes	MCI	MCI					No
480	PD	75	0.86	1.11	0.97				2	2	2		No	MCI	Dementia	Dementia	Dementia	Dementia	Dementia	Yes
481	PD	75	0.72	0.62	0.58				2	2	2		No	MCI	Normal	Normal	Normal	Normal		No

489	PD	55	0.70	0.84					1	2	2	2	No	Normal	Normal	Normal	Normal	Normal	Normal	No
490	PD	66	0.59	0.78					2	2	2		No	MCI	MCI	MCI	MCI	MCI	MCI	No
492	PD	70	0.71	0.73					2	2	2	2	No	MCI	MCI	MCI	MCI	MCI	MCI	No
493	PD	60	0.74	0.72					2.5	2.5	2	2	No	Normal	Normal	Normal	Normal	Normal	MCI	No
61	PSP	78	0.76	0.63	0.64	0.79			2	2	5		Yes	Normal	Normal					No
75	PSP	72	0.87	0.71	0.64	0.55			4	5	5		Yes	MCI	MCI	MCI	Dementia			Yes
88	PSP	74	0.72	0.75	0.75	0.76	0.72	0.78	2.5	3	4	5	Yes	MCI	MCI	MCI	MCI	MCI	Dementia	No
233	PSP	81	0.76	0.81	0.95	0.74	0.84		2.5	2.5	2.5		No	MCI	MCI	MCI	MCI	MCI	MCI	No
261	PSP	67	0.69	0.66	0.71	0.74	0.81		2	2	2		No	MCI	MCI	MCI	MCI	MCI	MCI	No
290	PSP	66	0.81	0.78	0.65	0.73			3	5	5		Yes	MCI	MCI	MCI	MCI			No
302	PSP	74	0.85	0.87	0.78	0.79	0.75		2.5	2	2		No	Normal	Normal	Normal	Normal	Normal	MCI	No
343	PSP	77	0.77		0.69	0.75			1.5		4		Yes	Normal	Normal	Normal	Dementia			Yes
358	PSP	80	0.65	0.73	0.57	0.59			3	3	3		Yes	Normal	Normal	Normal	Normal	Normal	MCI	No
363	PSP	84	0.56	0.63	0.74	0.64			4	4	4		Yes	Normal	Normal	Normal	MCI	MCI		No
404	PSP	86	0.73	0.62	0.74				5	4			Yes	MCI	MCI	MCI	MCI			No
406	PSP	64	0.77	0.68	0.78				2	2			No	MCI	MCI	MCI	Dementia	Dementia	Dementia	Yes
408	PSP	60	0.68	0.61					2	4			No	MCI	MCI	MCI	MCI	MCI	Dementia	No
416	PSP	73	1.19	0.93	1.07				3	4			Yes	MCI	MCI	MCI	Dementia			Yes
419	PSP	75	0.76	0.77	0.88				2	2			No	MCI	MCI	MCI	Dementia			Yes
421	PSP	79	0.55	0.55					2.5	2.5			No	MCI	Dementia					No
488	PSP	84	0.54	0.63					3	4			Yes	MCI	MCI					No
5	MSA	82				0.64					4		Yes	Normal	Normal	Normal	Normal			No
21	MSA	78	0.62	0.67	0.62	0.57	0.50		2	2	3		Yes	Normal	Normal	Normal	MCI	MCI	MCI	No
35	MSA	73	0.73	0.78	0.86		0.88	0.74	3	3		3	Yes	MCI	Normal	Normal	Normal	Normal	Normal	No
58	MSA	74	0.86	0.69	0.92	0.73			3	3	4		Yes	Normal	MCI	MCI	MCI			No
66	MSA	77	0.67						2.5				No	MCI						No
82	MSA	72	0.82	0.78	0.91	0.59	0.69	0.74	2	2	2	2	No	Normal	Normal	Normal	Normal	Normal	Normal	No
154	MSA	77	0.84	0.82	0.62		0.74		2	2.5			No	Normal	Normal	Normal	Normal			No
168	MSA	80	0.80	0.96	0.75	0.66			2.5	2.5	3		Yes	Normal	Normal	Normal	Normal			No
193	MSA	76	0.78	0.66		0.73	0.71		2.5	1	2.5		No	Normal	Normal	Normal	MCI	MCI	MCI	No
201	MSA	73	0.82	0.78	0.68				2	2			No	MCI	MCI	MCI	MCI			No
212	MSA	46	1.09						5				Yes	Normal						No
300	MSA	79	0.49	0.43	0.42	0.47			2	2	2		No	MCI	Normal	Normal	Normal	Normal	MCI	No
344	MSA	74	0.73	0.68	0.58	0.68			2	2	2		No	Normal	Normal	Normal	Normal	Normal	Normal	No
375	MSA	70		0.66		0.68				2	4		Yes	Normal	MCI	MCI	MCI	MCI	Dementia	No
463	MSA	88	0.78	0.84					3	3			Yes	Normal	Normal	Normal	MCI			No
83	control	72	0.66			0.53		0.55						Normal	Normal	Normal	Normal	Normal	Normal	No
84	control	71	0.80											Normal						No
85	control	69	0.76			0.80		0.70						Normal	Normal	Normal	Normal	Normal	Normal	No
90	control	75	0.96					0.85						Normal	Normal	Normal	Normal	Normal	Normal	No
91	control	67	0.62					0.65						MCI	Normal	Normal	Normal	Normal	MCI	No
92	control	73	0.79											Normal						No
93	control	76	0.83					0.74						MCI	MCI	MCI	MCI	MCI	MCI	No
94	control	66	0.88											Normal						No
95	control	69	0.94			0.84								Normal	Normal	Normal	Normal			No
98	control	68	0.88			0.87		0.83						Normal	Normal	Normal	Normal	Normal	MCI	No
99	control	67	0.85			0.74								MCI	MCI	MCI	MCI			No
102	control	72	0.67			0.68		0.68						Normal	Normal	Normal	Normal	Normal	Normal	No
104	control	74	0.68			0.81		0.64						MCI	MCI	MCI	Normal	Normal	MCI	No
116	control	68	0.74			0.74		0.74						Normal	Normal	Normal	Normal	Normal	MCI	No
117	control	69	0.73			0.75		0.70						Normal	Normal	Normal	Normal	Normal	Normal	No
123	control	71	0.97			0.79		0.86						Normal	Normal	Normal	Normal	Normal	Normal	No
124	control	66	0.94			0.91		0.91						Normal	Normal	Normal	Normal	Normal	Normal	No
125	control	72	1.01											Normal						No
126	control	70	0.80			0.86								Normal	Normal	Normal	Normal	Normal	Normal	No
127	control	71	0.86			0.82		1.01						Normal	Normal	Normal	Normal	Normal	Normal	No
128	control	76	0.76			0.62		0.64						Normal	Normal	Normal	Normal	Normal	Normal	No
142	control	71	0.71			0.65		0.61						Normal	Normal	Normal	Normal	Normal	Normal	No
144	control	75	0.92			0.96		0.86						Normal	Normal	Normal	Normal	Normal	Normal	No
145	control	78	0.81			0.72		0.71						Normal	Normal	Normal	Normal	Normal	Normal	No
179	control	64	0.68											Normal						No
192	control	52	0.92					0.89						Normal	Normal	Normal	Normal	Normal	Normal	No
200	control	57	0.70			0.74								Normal	Normal	Normal	Normal	Normal	Normal	No
242	control	48	1.02			0.83								Normal	Normal	Normal	Normal			No
244	control	63	0.87			0.66								Normal			MCI	MCI	MCI	No
260	control	61	0.87			0.71								Normal	Normal	Normal	Normal	Normal	MCI	No
